# The Feasibility of Smartwatch Micro–Ecological Momentary Assessment for Tracking Eating Patterns of Malaysian Children and Adolescents in the South-East Asian Community Observatory Child Health Update 2020: Cross-Sectional Study

**DOI:** 10.2196/73435

**Published:** 2026-02-06

**Authors:** Richard Lane, Louise A C Millard, Ruth Salway, Chris J Stone, Andy L Skinner, Sophia M Brady, Jeevitha Mariapun, Sutha Rajakumar, Amutha Ramadas, Hussein Rizal, Laura Johnson, Tin Tin Su, Miranda Elaine Glynis Armstrong

**Affiliations:** 1 Jean Golding Institute University of Bristol Bristol United Kingdom; 2 MRC Integrative Epidemiology Unit University of Bristol Bristol United Kingdom; 3 Population Health Sciences Bristol Medical School University of Bristol Bristol United Kingdom; 4 Integrative Cancer Epidemiology Programme School of Psychological Science University of Bristol Bristol United Kingdom; 5 Bristol Medical School Integrative Cancer Epidemiology Programme University of Bristol Bristol United Kingdom; 6 Population Health Sciences Institute Newcastle University Newcastle upon Tyne United Kingdom; 7 South East Asia Community Observatory (SEACO) Jeffrey Cheah School of Medicine and Health Sciences Monash University Malaysia Subang Jaya Malaysia; 8 Department for Health University of Bath Bath, England United Kingdom; 9 NIHR Bristol Biomedical Research Centre University Hospitals Bristol and Weston NHS Foundation Trust Bristol United Kingdom

**Keywords:** eating behavior, ecological momentary assessment, EMA, Malaysia, LMIC, children, adolescents, micro-interaction EMA, μEMA, smartwatch

## Abstract

**Background:**

Mobile phone ecological momentary assessment (EMA) methods are a well-established measure of eating and drinking behaviors, but compliance can be poor. Micro-EMA (μEMA), which collects information with a single tap response to brief questions on smartwatches, offers a novel application that may improve response rates. To our knowledge, there is no data evaluating μEMA to measure eating habits in children or in low-to-middle-income countries.

**Objective:**

In this study, we investigated the feasibility of micro-EMA to measure eating patterns in Malaysian children and adolescents.

**Methods:**

We invited 100 children and adolescents aged 7-18 years in Segamat, Malaysia, to participate in 2021-2022. Smartwatches were distributed to 83 children and adolescents who agreed to participate. Participants were asked to wear the smartwatch for 8 days and respond to 12 prompts per day, hourly, from 9AM to 8PM, asking for information on their meals, snacks, and drinks consumed. A questionnaire captured their experiences using the smartwatch and μEMA interface. Response rate (proportion of prompts responded to) assessed participants’ adherence. We explored associations between response rate with time of day, across days, age, and sex using multilevel binomial logistic regression modeling.

**Results:**

Eighty-two participants provided usable smartwatch data. The median number (IQR) of meals, drinks, and snacks per day was 2 (2-4), 3 (1-5), and 1 (0-2), respectively, on the first day of the study. The median response rate across the study was 68% (IQR 50-83). The response rate decreased across study days from 74% (68-78) on Day 1 to 40% (30-50) on Day 7 (odds ratio [OR] per study day 0.73, 95% CI 0.64-0.83). Response rate was lowest at the start of the day and highest between the hours of 12 PM and 2 PM. Female participants responded to more prompts than male participants (OR 1.72, 95% CI 1.03-2.86). There was no evidence of differential response by age (OR 0.73, 95% CI 0.41-1.28). Most participants (65%) rated their experience using the smartwatch positively, with 33% saying they were happy to participate in future studies using the smartwatch. For children that did not wear the smartwatch for the full study duration (n=22), discomfort was the most common complaint (41%).

**Conclusions:**

In this study of the feasibility of μEMA on smartwatches to measure eating in Malaysian children, we found the method was acceptable. However, response rates declined across study days, resulting in substantial missingness. Future studies (eg, through focus groups) should explore approaches to improving response to event prompts, trial alternative devices to increase children’s comfort, and evaluate revised protocols for reporting of intake events.

## Introduction

Noncommunicable diseases (NCDs) are the most common cause of death worldwide [1]. In Malaysia [2], NCDs particularly impact lower-income households [3]. Therefore, health surveillance in this population is required to better understand policy interventions that may improve health outcomes in Malaysia. Dietary risk factors accounted for 10% of all deaths globally in 2021 [4]; therefore, measuring eating is a crucial component of health surveillance. Traditional methods for measuring eating and dietary intake include food diaries, 24-hour recalls, diet histories, or food frequency questionnaires. While methods relying on memory of past behavior are subject to error like recall bias, prospective methods like diaries are affected by reactivity, where real or reported behavior is altered owing to the process of documenting food intake in real time. Underreporting is common in all existing methods, with an estimated 263 kcal per day typically missing from self-reported intakes compared with objective measures [5]. Underreporting varies with food type and eating occasion, with snacks and snack foods more likely to be left out of a self-reported record [6-9]. Online tool, such as Intake24 (Newcastle University), that guide users through a 24-hour recall process aim to reduce researcher burden in coding data collected. Photographic methods, where participants are asked to take pictures of their meals rather than write down each food and drink along with its portion size, aim to offer a more objective approach to add portion size estimation and reduced participant burden for capturing real-time food intake [10-12]. However, moving 24-hour recall online has not yet altered estimated underreporting [13,14], and issues with remembering to take photos before consuming foods as well as automating the estimation of foods and nutrients [15] in photos mean that outstanding challenges in dietary assessment methods remain [12]. Therefore, the feasibility of using alternative methods needs to be considered.

Ecological momentary assessment (EMA) is the repeated sampling of current behaviors in real-time in a natural environment [16]. EMA has evolved to be primarily delivered using mobile phones (mEMA), which have improved response rates compared with original pen and paper methods [17-19]. There is a large volume of literature on EMA using smartphones (n=796 studies) [20]. While diet is the second most commonly studied topic, it still only accounts for 4% (35/796) of these studies. Studies of diet using EMA in young people are primarily in the United States and Europe, with just 2 studies in Asia, in China, and Taiwan [18,21].

Liao [22] highlighted that response rates and compliance with EMA protocols were rarely reported. Since then, reporting of compliance has improved, but the response latency remains unknown from many studies [21]. Response rates to mEMA of diet are a median of 74% [23], which is similar to mEMA of all topics (mean 75% (IQR 64%-84%) [20]. A review of mEMA for diet in young people (16-30 years) showed response rates mostly exceeded 80% [21], whereas at younger ages poorer responses <80% are more often observed [18]. Lower response rates have also been associated with weekends versus weekdays [21], when participants receive more prompts during the day [17,20], and in males versus females [21].

Smartwatches are an emerging technology for collecting data alongside sensor data using micro-EMA (μEMA) protocols. This captures information using single-tap responses to brief questions, which is suitable for the small screens on these devices [19,23]. In adults, μEMA has been found to yield higher compliance rates despite more frequent sampling than mEMA and is perceived by users as less distracting [24]. Further, the use of μEMA significantly improves response rate (mean 72% vs 82%) but remains rare, with only 12 studies on any topic in any age group [20]. Despite these advantages, some limitations of μEMA have been reported in the literature, including limited battery life and technical problems such as problems with charging [25].

In children and adolescents, the use of pen and paper EMA to measure diet has typically been implemented outside of school hours [18]. Internet-connected devices such as mobile phones are often used for mEMA data collection [26]. These may be less suitable for child and adolescent populations, where 40% of education systems now ban the use of smartphones in school [27]. Further, devices such as smartwatches that can function without an internet connection may be better suited to rural, semirural, and low-resource settings where communication infrastructure may be less well-developed [28]. Therefore, smartwatches offer the potential to implement EMA across the whole day, with the potential for additional advantages such as improved compliance and response rates [20,24]. To our knowledge, only two diet studies involving adults in the United Kingdom and the United States have reported on EMA with smartwatches, and none have involved children [18,23,29].

Therefore, this study investigates the feasibility of using smartwatch-based μEMA to record eating patterns in Malaysian children and adolescents. The collected μEMA data are used to examine the completeness of the collected data and factors associated with response rates, alongside survey responses assessing participants’ experience during the study. Establishing the feasibility of this novel dietary measurement tool is an important first step to inform utility and any required refinement prior to deployment for dietary measurement more widely.

## Methods

### SEACO-CH20 Study

The South-East Asian Community Observatory (SEACO) health and demographic surveillance cohort is a dynamic cohort of 13,335 households in Segamat, a semirural region in the state of Johor Darul Takzim, Malaysia. The cohort was established in 2012, with surveys, blood tests, and physical measurement data collected from participants. In 2013 and 2018, health surveys were conducted on ~25,000 adults and children, 25,168 in 2013 and 24,710 in 2018.

All households (18,602) in 5 subdistricts, which SEACO operates, were invited to participate in the 2017 census. Altogether 11,617 households (40,184 residents) accepted our invitation. In 2018, participants who were involved in the 2017 census and were older than 5 years were invited to participate in the 2018 health round data survey, to which a total of 24,710 participants agreed. Potential participants from 3 subdistricts were preselected and approached via telephone using the existing health database (HR 2028). Participants’ parents were approached via telephone for recruitment before the home visit.

Children and adolescents aged 7-18 years who were part of the SEACO cohort were invited to participate in the SEACO Child Health 2020 update (SEACO-CH20) study; a systematic review of EMA studies in youth recommended 7 as a lower age limit for EMA [26]. The eligibility of households was limited by location due to the safety measures implemented during the COVID-19 pandemic to reduce the risk to participants, households, and fieldworkers. Therefore, the 1993 children and adolescents invited to participate were from only 3 of the 5 SEACO subdistricts (Jabi, Sungai Segamat, and Gemereh) in the Segamat district.

Data collection visits to individual households were performed in person from November 1, 2021, to July 31, 2022. The data were collected as part of a larger study and included surveys, physical measurements, such as height, weight, blood pressure, waist and hip circumference, and blood sample collection. Participants were given wrist-worn Axivity AX6 6-axis accelerometers to monitor their physical activity, which were worn 24 hours per day over 7 days [30]. A random subset of the participants were also given TicWatch C2 (Mobvo) Android smartwatches to record eating and drinking with μEMA as part of this feasibility study, using a smartwatch μEMA system developed within the research team. The smartwatch system used was an adaptation of a µEMA system first used in a study involving high-temporal density longitudinal measurement of alcohol use [31] by a subset of the research team who developed this system. Participants wore these devices over the same 7-day period as the accelerometers. As they are both wrist-worn devices, this may have affected the acceptability of the smartwatch. Participants were briefed on the use of these devices by the data collectors, including how to charge them and how to replace them after charging. The original data collection plan can be seen in Figure S1 in Multimedia Appendix 1. A completed Checklist for Reporting Ecological Momentary Assessment Studies (CREMAS) [22] can be found in Multimedia Appendix 2.

### Study Participants

Participants for the SEACO-CH20 study were selected from the larger SEACO cohort. Parents of participants provided consent for their children to participate in SEACO-CH20. A random subsample of 100 participants were each invited to wear a smartwatch.

### Data Collection

SEACO-CH20 fieldworkers performed 2 home visits to collect the data. The smartwatch was distributed on the initial visit, and the participants were briefed on how to use and charge it. They were instructed to wear the device for the next 8 days, on “the wrist that [they] use to write.” The smartwatches were collected during the second home visit, and the participants were asked to complete a questionnaire on their experience with the devices. Questionnaires assessed the participants’ attitudes toward several aspects of the smartwatch study, including ease of use, their attitude toward charging, and their overall experience. Since children as young as 10 were asked to complete the questionnaires, a reduced set of acceptance questions was used to reduce burden. This was based on similar pilot work using novel methods in the ALSPAC G2 study [32] and included the following questions:

- Overall, how would you rate your experience of using the smartwatch during the study, on a scale from 1 (didn’t like it at all) to 5 (really liked it)?
- If you were asked to use the smartwatch again in another study, would you participate?
- How many days in total did you wear the smartwatch for?
- If you wore the smartwatch for less than 8 days, what were the main reasons for not wearing it longer?

Parents of participants aged 7-9 years completed the survey on behalf of their children, while participants aged 10 years and older filled out the survey themselves. The full text of the survey can be found in Figure S2 in Multimedia Appendix 1.

### Smartwatch μEMA Questions

During the study, the smartwatch prompted participants once every hour to enter any food or drink that they had consumed in the last hour. These prompts were scheduled to appear once every hour from 9 AM to 8 PM, so participants were expected to interact with the smartwatch 12 times throughout the day. We chose this hourly prompt frequency to maximize the chance that eating and drinking events were less likely to be missed and to capture more fine-grained temporal patterns in eating behavior. As this was a feasibility study, this choice was justified, given that μEMA has been shown to improve compliance despite more prompts than mEMA in adults [26]. The smartwatch interface included the following 5 questions that the participants completed for each item consumed:

1. Have you had any food or drink in the last hour? Options: yes, no
2. What did you have? Options: meal, snack, drink
3. What size was it? Options: small, medium, large
4. What did you use to eat? Options: hands, fork/spoon, chopsticks
5. Where were you? Options: home, school, elsewhere

A possible future use of this methodology, once fully refined, could include automated eating detection [33]. Therefore, we included a question on the type of cutlery used (“What did you use to eat?”), as lack of information on utensil type has been highlighted as a limitation of some datasets used for algorithm development related to automated eating detection [34].

After entering this information for one item consumed, they were asked, “Any more food or drink to record?” and could then start again to add another entry. Therefore, each consumption entry either indicates that the participant did not eat or drink in the last hour or contains the answers to the above questions for a particular meal, drink, or snack, linked to an hour period within a day. If participants ignored the prompts, they would receive a reminder prompt after 1 minute; if they continued to ignore the prompt for a further 1 minute, the prompt would disappear and “no response” would be recorded by the smartwatch.

Participants could choose “back” on each question screen to return to a previous question and update their response. However, after submitting their answers for a particular item (ie, completing the “where were you” question for that item), they would not be able to return to that entry.

An additional prompt (the “catch-up”) was scheduled every morning at 8 AM asking if they had consumed any food or drink on the previous day that had not been recorded on the smartwatch. If they indicated “yes,” they were asked the same questions as above. Catch-up entries did not have an associated eating time but were labeled as catch-up–type events, indicating that they applied to the previous day.

The smartwatch study was co-created and piloted with the Malaysian research team and the English was translated into Malay for use on the smartwatch. All the original data collection was in Malay. The smartwatch protocol, including the prompts and possible responses, can be seen in Tables S1-S3 in Multimedia Appendix 1.

### Smartwatch Data Cleaning

Smartwatches were distributed by fieldworkers partway through the day, and μEMA responses on this distribution day were removed from analyses. The study period is taken to be the subsequent 7 days after this distribution day.

The version of the EMA software we used did not save the hour period to which each entry belonged. Therefore, we needed to infer this from the submission timestamp, the date and time a particular entry was submitted. As entries for the same hour period are submitted one after the other, we used a time window to group nearby entries into a single “eating event,” which is intended to capture the participants’ responses to one prompt. A 30-minute window was chosen to group nearby prompts, as we expect this to collect entries from the same eating event without grouping prompts from adjacent hours. Previous work from diet diaries suggests that 30 minutes is a reasonable cut-point to distinguish independent eating occasions [35]. Occasionally, there may be participants with more than 12 eating events per day, for example, if they took more than 30 minutes to finish responding to a prompt. This occurred on 26 occasions, less than 5% of the total 574 (82 participants multiplied by 7 days) study days.

The μEMA data used was restricted to the 7 days after the distribution day. During the data review, we identified an issue with the collected data where there were sometimes multiple identical entries for a given intake event due to an issue with the μEMA software. Therefore, duplicate entries were identified as any pair of entries with identical contents (same meal type, portion size, utensil, and location), for the same hour period, and entered within 5 minutes of each other. The first such entry was kept in each case. Around 588 duplicate responses were removed of 10,539. Data cleaning was performed in Python (version 3.10.0; Python Software Foundation).

### Response Rate

The response rate was calculated as the proportion of prompts responded to (with either at least one item consumed or an entry stating that they did not eat or drink anything in the previous hour). The response rate tells us the extent that participants engaged with the smartwatch app throughout the day but not the extent that the data entered are complete, that is, whether all intake events were recorded. We therefore summarized the number of each type of meal entry (meal, drink, snack, or no food or drink) submitted per day across our sample. For these summaries we included only participants who took part in the study outside the Ramadan fasting period (April 3, 2022-May 1, 2022; n=67), since fasting participants are likely to enter fewer eating events during the day. The mean of participants’ response rates per day was recorded, and the median and quartiles of these were reported.

Attrition from the study was examined by identifying the last day each participant responded to any smartwatch prompt; participants who had ceased responding to the prompts are referred to as “inactive.”

### Statistical Analyses

We summarized response rates to each individual prompt of the smartwatch μEMA and the participants’ experiences based on the survey questions. We used mean for continuous variables, n (%) for categorical variables, and median and IQR for ordinal or nonnormally distributed continuous variables.

We used a mixed-effect logistic regression model for the response (yes or no) to an individual prompt on a specific study day of data collection for each participant. A fixed effect term was included for study day (from the first to the seventh day) as a continuous linear trend. The time of day was also included as a fixed effect to capture nonlinearity in response throughout the day, grouping the prompts by the nearest hour as follows to decrease the number of parameters in the model:

- Morning (9-11 AM)
- Lunchtime (12-2 PM)
- Afternoon (3-5 PM)
- Evening (6-8 PM)

Random intercept and random slope terms were included for study day within each participant. Estimates are provided as odds ratios (OR) and 95% CIs, interpreted as the multiplicative change in the odds of a participant responding to an individual prompt. The degree of difference between participants was summarized in the intraclass correlation coefficient.

To evaluate if changes in participation across wear days differed in boys versus girls, we repeated this base model, adding a fixed term for sex and an interaction term between sex and study day. Similarly, we explored differences by age group (Malaysian primary school age: 7-12 years versus secondary school age: 13-18 years) by adding a fixed term for age group and an interaction term between age group and study day to the base model.

Analyses were performed in Python version 3.10.0 and R (version 4.2.2; R Core Team) [36]. All of our analysis code is publicly available [37]. Git tag 3.0 (The Git Project) corresponds to the version of the analyses presented here.

### Ethical Considerations

Written informed consent was obtained from parents or guardians on behalf of the participants. Children and adolescents were also asked to provide their written assent to participate in the study. Ethical approval was obtained from the Monash University Human Research Ethics Committee on March 17, 2020 (Project ID: 23271) and the University of Bristol REC Case no. 2020–4208 (ID nr: 1304255) prior to any data collection. The study was conducted in accordance with the Declaration of Helsinki for experiments involving humans.

Participants were given a token worth up to RM25 to compensate for their time participating in the study. This was divided into RM5 for completion of each of the following components: (1) questionnaires, (2) health check, (3) blood sample, (4) activity monitor, and (5) smartwatch. They were also given a free health screen and a direct referral to the government primary health care clinic if they were identified as high risk.

Study data have been deidentified and can be freely requested from SEACO, Monash University Malaysia Institutional Data Access at “mum.seaco@monash.edu” for researchers meeting the criteria for access to confidential data. Please refer to the web resource hosted on Monash University’s website [38] for more information.

## Results

### Participants

**Figure 1:**
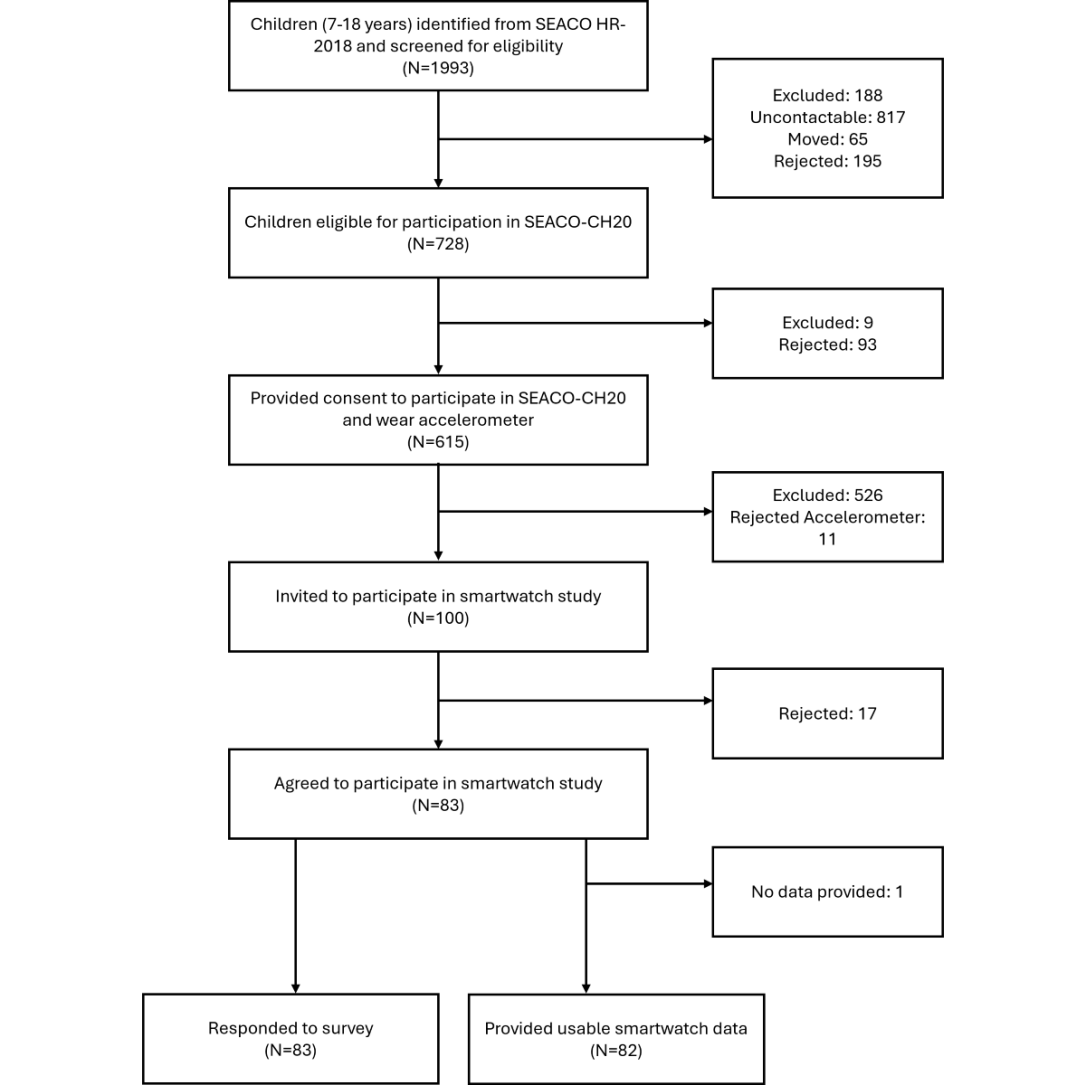
Study flowchart. Eligible participants were selected from the SEACO Health Round Survey 2018 (SEACO HR-2018) cohort. Reasons for rejecting the smartwatch study included concern about discomfort and allergies. SEACO-CH20: South-East Asian Community Observatory Child Health 2020; SEACO HR-2018: South-East Asian Community Observatory Health Round Survey 2018;.

A flowchart showing the study participants can be seen in Figure 1. Parents of 728 participants consented to their children’s participation in SEACO-CH20, of which 626 provided demographic (age, sex, and ethnicity) and accelerometer data for the larger study. Of these, 100 participants were randomly invited to wear a smartwatch for this smaller feasibility study. Of the 100 participants invited to participate in the smartwatch substudy, 83 participants agreed. The reasons for nonparticipation included concern the device was not comfortable (n=3) and allergies (n=2). The remaining participants rejected the smartwatch without comment. One further participant accepted the smartwatch study but removed the EMA app from the watch during the study period, rendering their data unrecoverable, resulting in 82 participants who provided smartwatch data.

The sex, ethnicity, and age breakdown for all participants who took part in the smartwatch study can be seen in Table 1.

**Table 1:** Summary of participant demographics (N=83).

Participant characteristic			Smartwatch participants, n (%)
**Sex**			
	Female	53 (64)	
	Male	30 (36)	
**Ethnicity**			
	Malay	73 (88)	
	Non-Malay	10 (12)	
**Age (years)**			
	7-12	24 (29)	
	13-15	28 (34)	
	16-18	31 (37)	

### Smartwatch Responses

The median prompt response rate was 69% (IQR 52%-82%).

**Figure 2:**
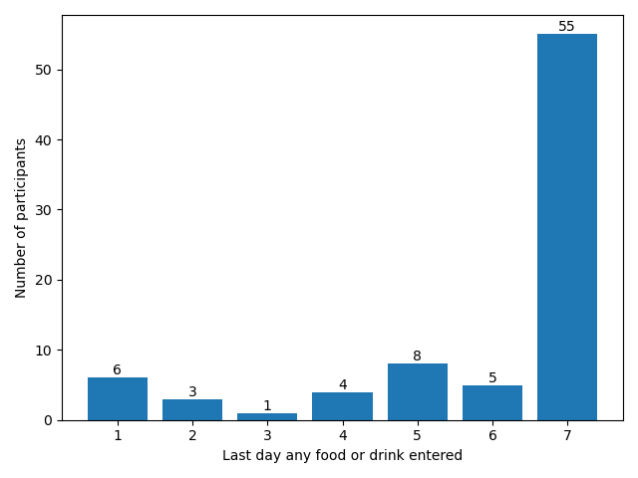
The number of participants who became inactive on each day (N=82), that is, having responded to no μEMA prompts after this day. All participants were active for at least one day.

The number of participants who became inactive on each day can be seen in Figure 2. The majority (55/82, 67%) of participants were active until day 7, that is, they responded to at least 1 prompt on day 7.

The median and IQR in the number of entries of each type made by each participant per day are summarized in Table 2. Fifteen participants took part (at least partially) during Ramadan and so were excluded from these summaries. Only a minority of intake events were submitted as catch-up entries (N=125 catch-up entries versus 4705 noncatch-up).

**Table 2:** The median and IQR of the number of noncatch-up entries per day per participant, for participants whose study period did not intersect with Ramadan (N=67).

Study day	Meal, median (IQR)	Drink, median (IQR)	Snack, median (IQR)
1	2 (2-4)	3 (1-5)	1 (0-2)
2	2 (1-3)	2 (1-5)	1 (0-2)
3	2 (1-3)	2 (0-3)	0 (0-1)
4	2 (1-3)	1 (0-3)	1 (0-1)
5	1 (0-2)	1 (0-3)	0 (0-1)
6	1 (0-2)	1 (0-2)	0 (0-1)
7	1 (0-2)	0 (0-2)	0 (0-1)

### Response Rate Across and Within Study Days

The response rate for individual prompts had a median (IQR) of 67% (50-83). The response rate on each day ranged from 83% (66-92) on day 1 to 58% (33-75) on day 7.

**Figure 3:**
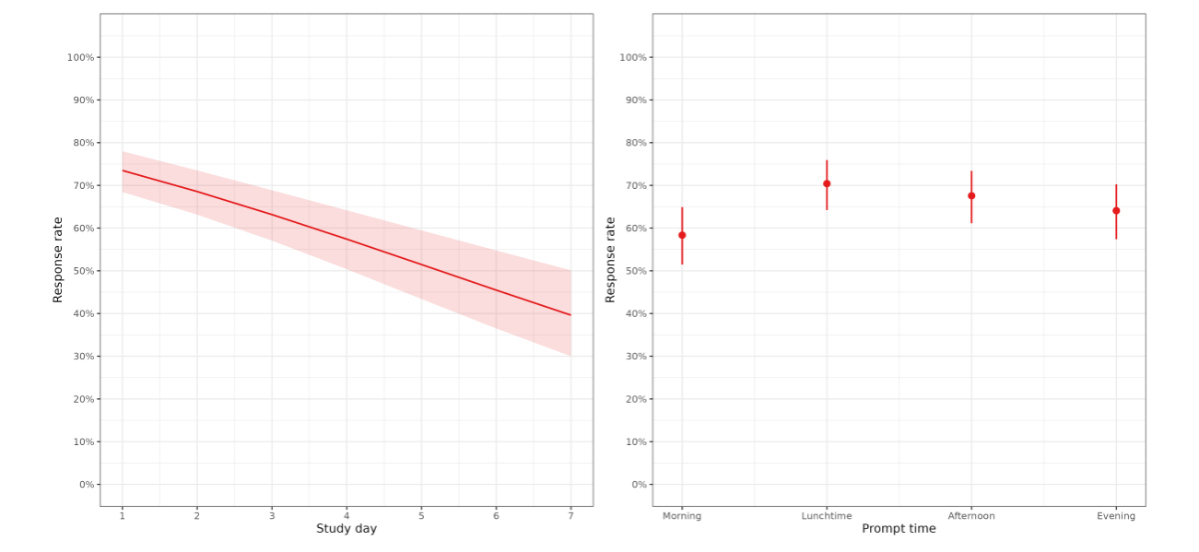
Participants’ response rates against study day (left) and time of day (right; N=82).

Figure 3 shows the response rate with study day and time. The response rate decreased across study days (OR for each additional day of the study: 0.73 (95% CI 0.64-0.83). The response rate was lowest at the beginning of the day; the OR and 95% CIs are summarized in Table 3. The intraclass correlation coefficient was 0.207, which indicates that approximately 21% of the total variance in prompt response behavior was attributable to between-participant differences.

**Table 3:** The odds ratios for responses at different times of day, taking breakfast time (9-11AM) as the reference level. The response rate was lowest at the beginning of the day.

Time	Odds ratio (95% CI)
Breakfast (9-11 AM)	1^a^ (0-0)
Lunchtime (12-2 PM)	1.69 (1.43-2.12)
Afternoon (3-5 PM)	1.49 (1.25-1.76)
Evening (6-8 PM)	1.27 (1.07-1.51)

^a^Reference level.

**Figure 4:**
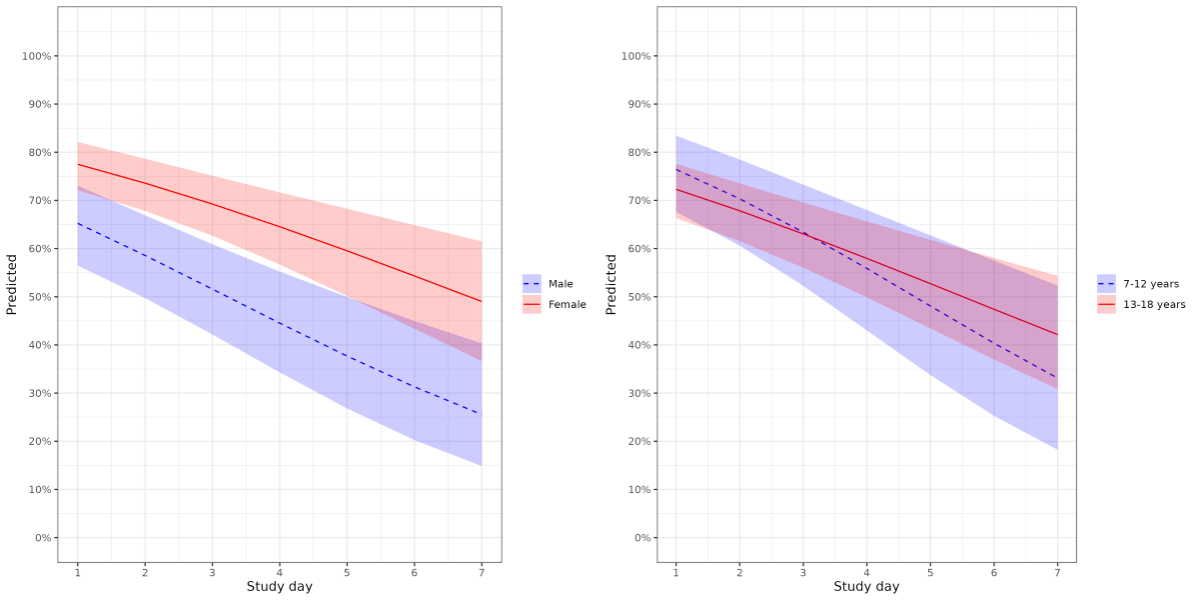
Association of micro-interaction ecological momentary assessment (μEMA) prompt response with study day stratified by sex (left) and age (right).

The results of analyses estimating differences due to sex and age are shown in Figure 4. Girls responded more often to the μEMA prompts compared with boys (OR 1.71, 95% CI 1.03-2.84). However, the daily patterns were similar for both sexes (interaction term OR 1.07, 95% CI 0.93-1.23). Response rate did not differ between age groups (OR 0.73, 95% CI 0.42-1.27), and daily response patterns were similar for the 2 age groups (study day-by-age interaction OR 1.11, 95% CI 0.95-1.29).

### Evaluating Participants’ Survey Responses on Acceptability

A summary of responses to questions about smartwatch acceptability and wear time is provided in Table 4.

A total of 54 out of 83 (65%) participants rated their experience using the smartwatch positively (a rating of 4 or 5 out of 5), 20 out of 83 (24%) gave a neutral rating (3 out of 5), and 8 out of 83 (10%) rated it negatively (1 or 2 out of 5). In addition, 27 out of 83 (33%) participants said that they would be happy to participate in future studies using the smartwatch, while 39 out of 83 (47%) said maybe, and 12 out of 83 (14%) said no. The majority of participants who responded (61/83, 73.5%) reported wearing the smartwatch for the entire duration of the study (8 days).

**Table 4:** The survey questions. Participants were directed to wear the smartwatch on the day that the watch was distributed and for the 7 subsequent days, making 8 days total. Missing data and participants who refused to respond are not included (N=83).

Question and response			Participants, n (%)
**Overall, how would you rate your experience of using the smartwatch during the study, on a scale from 1 (didn’t like it at all) to 5 (really liked it)?**			
	Negative (1 or 2)	8 (10)	
	Neutral (3)	20 (24)	
	Positive (4 or 5)	54 (65)	
**If you were asked to use the smartwatch again in another study, would you participate?**			
	No	12 (14)	
	Maybe	39 (47)	
	Yes	27 (33)	
**How many days in total did you wear the smartwatch for?**			
	5 or fewer	7 (9)	
	6	7 (8)	
	7	8 (10)	
	8	61 (73)	

For those who reported that they did not wear the smartwatch for the entire duration of the study (22 participants), the most common reason was that they did not find it comfortable to wear (9/22, 41%). Other reasons included forgetting, that they did not see the benefit when they could not see the data, they were forbidden to wear it by school, and it ran out of battery (all 3 or fewer responses).

Summaries of the participants’ responses to the remaining survey questions can be found in Tables S4-S8 and Figure S3 in Multimedia Appendix 1. Further summary statistics, including catch-up events and participants who took part during Ramadan, can be found in Tables S9-S11 in Multimedia Appendix 1.

## Discussion

### Principal Findings

In this feasibility study of a smartwatch-based μEMA method to collect data on eating habits over 7 days in Malaysian children, we found that most participants (55/83, 67%) remained responsive to prompts up to the last day of the study. Participants were least likely to respond to prompts between 9 and 11 AM and most likely between 12 and 2 PM. The intraclass correlation coefficient was 20.7%, suggesting that while some variation in response pattern is attributable to between-participant differences, the majority of the variation (79.3%) was due to within-participant differences. The response rate dropped off day-on-day and was higher for female than male participants; no association was found between participant age group and response rate.

Our average response rate of 69% was lower than the average of 78% found in a meta-analysis of EMA in children and adolescents, including studies that prompted between 2 and 9 times daily [17]. That study found that prompting participants more often had a large negative effect on completion rate, which is further supported by Kraft et al [20], which found a negative correlation of –0.12 between increased number of prompts and response rate (*P*=.009). Participants in our study were prompted 12 times a day, plus an additional catch-up prompt in the morning. We justified our original prompt frequency choice, as μEMA has been shown in adults to improve compliance despite a higher number of prompts than mEMA. However, in our study using a child and adolescent population, it is likely a higher prompt frequency may have had a negative effect on response rate, especially in the case of repeated “No food/drink” entries. It has been reported [17] in nonclinical studies that “a higher average compliance rate was observed in studies that prompted participants 2-3 times daily (91.7%) compared with those that prompted participants more frequently (4-5 times: 77.4%; 6+ times: 75.0%).” This suggests that compliance may be improved by prompting participants less frequently, for example, by having 3 prompts daily at 11 AM, 3 PM, and 7 PM, although longer time intervals increase the reliance on memory, potentially affecting the completeness of recorded consumption events. It is also possible that our lower response rate might have been related to the number of questions asked in our μEMA protocol. A study that compared the deployment of 6 back-to-back multiple-choice questions delivered via a smartwatch versus a mobile phone found no difference in compliance between these 2 modalities. However, compliance was improved when single questions requiring a one-touch response were asked via a smartwatch, despite an increase in prompt frequency [39]. While on average, the participants (65%) rated the study protocol positively (either 4 or 5 out of 5), the response rate fell day-on-day. Participants were less likely to respond to prompts at the beginning or end of the day, compared with the middle of the day. Focus group or interview discussions were not feasible in this study due to COVID-19 restrictions but should be explored in future studies to determine the reasons for missing event prompts and nonresponses, which may include forgetting or being involved in a competing activity when the prompt is sent [40].

Female participants had a higher response rate than male participants, consistent with previous findings [21,41]. There was little evidence of difference in the relationship between response and study day for male versus female participants. An analysis of the SEACO-CH20 accelerometer dataset [30] found that a similar proportion of males and females had usable accelerometer data, suggesting that this was specific to the smartwatches rather than a difference with wrist-worn devices generally. Little evidence of a difference was found between response rates in the 7-12 years old and 13-18 years old age groups.

Although the subjective indicators suggested that most participants enjoyed wearing the smartwatch, only a minority of participants (27/83, 33%) indicated that they would be willing to participate in a similar study again; 39 out of 83 (47%) responded “Maybe.” Potential changes to the protocol that may improve compliance could include only wearing the smartwatch instead of both the smartwatch and accelerometer.

### Strengths and Limitations

This is the first study exploring the feasibility of using smartwatch-based EMA in a population of children and adolescents from a low-to-middle-income country. This study was part of the SEACO study, using the SEACO-CH20 dataset, and lays the foundation for an improved understanding of the potential for wearable devices for measuring relationships between eating and cardiometabolic health. Data on 24-hour eating behaviors are important for informing future policy that may reduce cardiometabolic risk among children and adolescents and prevent progression to cardiometabolic disease in adulthood. While a food frequency questionnaire was completed as part of the larger SEACO-CH20 study and reported elsewhere [42], this current study has assessed an alternative for recording behavior in real time.

However, this study did have some limitations. The number of questions in prompts may have affected compliance and should be discussed with participants to optimize the protocol for future studies in this population. It has been suggested [17] that compliance can be improved by incentivizing participants with a monetary reward or raffle entries. Since this study concerns young people, one potential incentive method could be to gamify the EMA process using a level-up or promotion system in the app [43]. Previous studies have explored adding an end-of-day catch-up prompt, which has been found to improve the reporting of dinner [40]. Replacing the morning catch-up prompt with one in the evening may improve response rate, especially given that we found that participants were more likely to respond to prompts in the evening than in the morning. Future studies may additionally consider using the catch-up entries to impute missed entries on the previous day, which could give more complete data. Another suggestion could be to incorporate a short period of training to improve response rates, where responses are monitored in real time by researchers and participants are prompted directly by researchers if missing responses are common. Such an approach has been used to improve the accuracy of real-time food photography methods [12].

The questionnaires used for acceptability and acceptance were not standard because we were motivated to use a reduced set of questions (that have been used in a previous publication [31]) to reduce the burden on the young participants. Future studies should consider if the emerging, more standard approaches to exploring acceptability and acceptance for wearable devices (eg, those based on Technology Acceptance Models) have been developed to the point at which varying levels of participant burden can be accommodated.

Unbalanced statistics limited our ability to assess differences across age, sex, and ethnic group. The larger SEACO-CH20 accelerometer study [34] from which participants for this study were selected had more balanced statistics (49% female, 67% Malay, and 44% <13 years old), which suggests that the cohort used for smartwatch data may not represent the overall SEACO-CH20 cohort. In particular, we only had 4 participants aged 7-9 years, so further studies are required to better understand the feasibility of dietary μEMA in the younger participants. Participants in this study began wearing the devices on different days of the week, and it is possible that the day of the week could affect participation; for example, whether it is a weekend or weekday. A lower response rate on weekends has been previously documented by Battaglia [21]. We did not account for study start day due to the small sample size, and because schooling was disrupted throughout the study period due to the COVID-19 pandemic [44].

An issue with entry duplication meant that some entries may have been removed that were actual events, not due to the software issue. This bug with the smartwatch software has since been fixed. Additionally, only the response time of the participant was recorded, and not the time that the prompt was sent. This means we had to infer which hour window the entries corresponded to; this could be programmed in the software.

Discomfort was the most common reason for nonwear cited by the participants, which may be unique to our study protocol that required participants to wear 2 wrist-worn devices on the same arm. Furthermore, the smartwatch used in our study was not specifically designed to fit smaller children. Efforts to make smartwatches less intrusive, for example, by making them smaller, may further improve response rate and study uptake.

Ramadan, a culturally important event in Malaysian society, which includes fasting in some population groups, took place during the course of the data collection period. This is likely to have affected the eating behaviors of participants who took part during this time. To ensure that the number of EMA entries was not influenced by Ramadan, we excluded participants from our analyses who wore the smartwatch during the Ramadan period, thereby further limiting our sample size.

### Conclusions

This study extends previous eating behavior studies by exploring the use of μEMA in a population of children and adolescents in Malaysia and is the first such study to do so. Willingness to take part in the μEMA study was high, but poor response rates suggest that the number of questions asked per prompt or the high number of prompts per day may be too burdensome. While our smartwatch-based EMA app was largely based on the μEMA methods originally developed by Intille et al [24], a key aspect of true μEMA implementation is the presentation of only one question at a time. In our approach, we chained questions to capture details on food and drink type, size, and consumption context, making it more accurately described as a modified μEMA. Further work is needed to explore different μEMA variations, including using fewer questions and/or fewer prompts, and identify devices that may be more comfortable for child and adolescent participants. The growing use of smartwatches amongst children, particularly in Southeast Asia may offer more opportunities for further study [45].

## Appendix

Multimedia Appendix 1 Supplementary information, including the original data collection plan; survey instruments; smartwatch EMA protocols and prompts; and additional smartwatch and survey tables and figures.

Multimedia Appendix 2 Checklist for Reporting Ecological Momentary Assessment Studies (CREMAS).

## Abbreviations

CREMAS: Checklist for Reporting Ecological Momentary Assessment Studies
EMA: ecological momentary assessment
mEMA: mobile ecological momentary assessment
NCD: noncommunicable disease
OR: odds ratio
SEACO: South-East Asian Community Observatory
SEACO-CH20: South-East Asian Community Observatory Child Health 2020
μEMA: micro-interaction ecological momentary assessment

## Data Availability

Data cannot be shared publicly for confidentiality and ethical reasons. Deidentified data are available and can be freely requested from the South East Asia Community Observatory, Monash University Malaysia Institutional Data Access at “mum.seaco@monash.edu” for researchers who meet the criteria for access to confidential data. For more information, please refer to [38].
