# Supplementary material for: The Feasibility of Smartwatch Micro–Ecological Momentary Assessment for Tracking Eating Patterns of Malaysian Children and Adolescents in the South-East Asian Community Observatory Child Health Update 2020: Cross-Sectional Study
Source: J Med Internet Res. 2026 Feb 6;28:e73435. doi: 10.2196/73435 (PMC12924041; doi:10.2196/73435)
Supplement: Multimedia Appendix 1 [file jmir_v28i1e73435_app1.doc]

# Supplementary material

## Methods

### Data Collection

A flow chart of the original data collection plan can be seen below.

*Figure S1 - the data collection plan.*

### Survey

This section contains the text of the questions asked to participants (Figure S2).


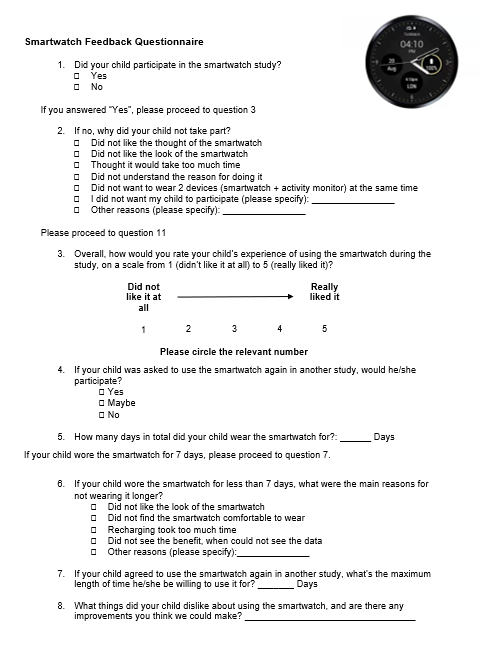


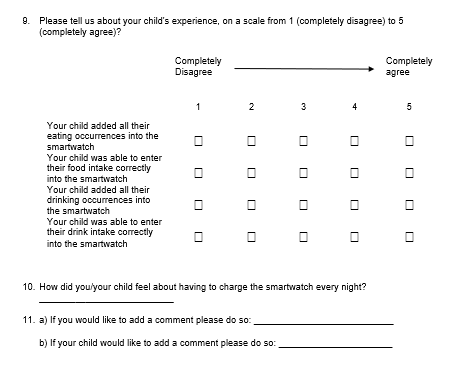


Figure S2 - the smartwatch survey. This version was given to the parents of the younger children; older children filled out their own surveys.

### Smartwatch Protocol

The EMA prompts and responses are summarised in the below table.

Table S1 - the EMA protocol.

| ***Prompt no.*** | ***Prompt and response options*** | ***Next prompt*** |
| --- | --- | --- |
| **0 (start of day)** | **Any food or drink you forgot yesterday?** |  |
|  | No (no food or drink unrecorded from yesterday) | 1 |
|  | Yes (food or drink to be added from yesterday) | 2 |
| **1** | **Have you had any food or drink in the last hour?** |  |
|  | Yes (food or drink consumed in last hour) | 2 |
|  | No (no food or drink consumed in last hour) | 1 (after 1 hour) |
| **2** | **What did you have?** |  |
|  | Meal | 3a |
|  | Snack | 3a |
|  | Drink | 3b |
|  | *Go back to “Did you have any food or drink in the last hour?”* | *1* |
| **EITHER 3a** | **What size was it?** |  |
|  | Small | 4 |
|  | Mid-size | 4 |
|  | Large | 4 |
|  | *Go back to “What did you have?”* | *2* |
| **OR 3b** | **What size was it?** |  |
|  | Small | 5 |
|  | Mid-size | 5 |
|  | Large | 5 |
|  | *Go back to “What did you have?”* | *2* |
| **4** | **What did you use to eat?** |  |
|  | Hands | 5 |
|  | Fork/spoon | 5 |
|  | Chopsticks | 5 |
|  | *Go back to “What size was it?”* | *3* |
| **5** | **Where were you?** |  |
|  | Home | 6 |
|  | School | 6 |
|  | Elsewhere | 6 |
|  | *Go back to “What did you have?”* | *2* |
| **6** | **Any more food or drink to record?** |  |
|  | No (no more food or drink to record for this hour) | 1 (after 1 hour) |
|  | Yes (more food or drink to record for this hour) | 2 |

### Smartwatch Prompts

The prompts sent to the smartwatches can be seen in Supplementary Tables 2 and 3.

The regular prompts were worded as follows:

Table S2 - the regular prompts sent to the smartwatches.

| ***English*** | ***Malay*** |
| --- | --- |
| **Have you had any food or drink in the last hour?** | **Adakah anda makan/minum pada jam sebelumnya?** |
| Yes (food or drink consumed in last hour) | Ya |
| No (no food or drink consumed in last hour) | Tidak |
| **What did you have?** | **Apa yang anda ambil?** |
| Meal | Makanan |
| Snack | Snek |
| Drink | Minuman |
| *Back* | *Kembali* |
| **What size was it?** | **Apa saiz yang diambil?** |
| Small | Kecil |
| Mid-size | Sederhana |
| Large | Besar |
| *Back* | *Kembali* |
| **What did you use to eat?** | **Apa yang anda guna?** |
| Hands | Tangan |
| Fork/spoon | Sudu/Garfu |
| Chopsticks | Chopstick |
| *Back* | *Kembali* |
| **Where were you?** | **Di mana anda ketika itu?** |
| Home | Rumah |
| School | Sekolah |
| Elsewhere | Lain-lain |
| *Back* | *Kembali* |
| **Any more food or drink to record?** | **Adakah anda ada makan atau minum yang lain?** |
| No | Tidak |
| Yes | Ya |

The wording of the catch-up prompts was:

Table S3 - the catch-up prompts sent to the smartwatches.

| ***English*** | ***Malay*** |
| --- | --- |
| **Any food or drink you forgot yesterday?** | **Anda ada lupa rakam makan atau minuman semalam?** |
| No (no food or drink unrecorded from yesterday) | Tidak |
| Yes (food or drink to be added from yesterday) | Ya |

## Results

### Survey

*If you wore the smartwatch for less than 7 days, what were the main reasons for not wearing it longer?*

Six adolescents responded “Other” to this question and gave additional explanations:

*Table S4* - reasons that participants did not wear the watch for the duration of the study.

| Malay | English |
| --- | --- |
| Lupa | Forget |
| Terlupa | Forgotten |
| Terlupa pkai selepas aktiviti merenang | Forgot to use after swimming activity |
| Pihak sekolah tidak mambenarkan memakai gajet | The school does not allow the use of gadgets |
| Kahabisan bateri | Out of battery |
| Sakit dan gatal | Pain and itching |

#### If you agreed to use the smartwatch again in another study, what is the maximum length of time that you would be willing to use it for?

A bar chart of participants’ responses can be seen in Figure . The plurality of participants indicated that they would wear it for 7 or 8 days at most. The majority of participants indicated that they would wear it for at least as long as they had in this study. 8 participants refused to answer.


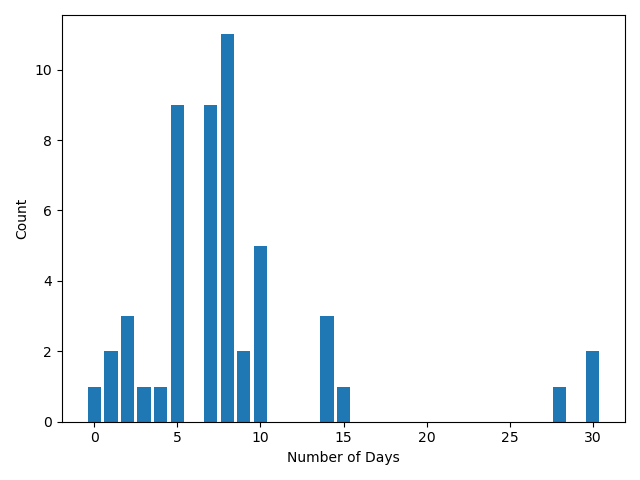


*Figure S3 - the number of days that participants reported they would wear the smartwatch for in another study (N=59).*

#### What things did you dislike about the smartwatch, and what improvements do you think we could make?

Most participants provided no data in response to this question. Of those who did, their responses are summarised in Table S55.

*Table S5* - what did you dislike about the smartwatch?

| Adolescent Response (Malay) | Response (English) | Count |
| --- | --- | --- |
| No | Tiada/Tidak | 7 |
| Writing | Semasa menulis/Tulis | 2 |
| 6 hours a day | 6 JAM SEHARI | 1 |
| Jangan pakai waktu bersenam | Don’t use exercise time | 1 |
| Tanya soalan yang sama | Ask the same question | 1 |
| Meletakkan corak | Placing the pattern | 1 |
| Berat dan mudah retak | Heavy and easy to crack | 1 |
| Ada penambaikan di jam | There are improvements in the clock | 1 |

#### Please tell us about your experience, on a scale of 1 to 5?

The subjective experience for some aspects of the study are summarised in Supplementary Table 6Error: Reference source not found. The plurality of participants did not respond to these questions; of those who did, the majority felt that they entered all their eating occurrences and that they entered them correctly (either 4 or 5 on the scale). A large proportion of participants did not respond to this question, potentially indicating survey fatigue.

*Table S6 - responses to questions on their experience using the smartwatches (N=63).*

| Question | Response | N | % |
| --- | --- | --- | --- |
| 1. You added all your eating occurrences into the smartwatch | 1 (Completely disagree)  2 | 4 | 7 |
|  | 3 | 17 | 28 |
|  | 4 | 18 | 30 |
|  | 5 (Completely agree) | 22 | 36 |
| 1. You were able to enter your food intake correctly into the smartwatch | 1 (Completely disagree)  2 | 5 | 9 |
|  | 3 | 14 | 24 |
|  | 4 | 14 | 24 |
|  | 5 (Completely agree) | 25 | 43 |
| 1. You added all your drinking occurrences into the smartwatch | 1 (Completely disagree)  2 | 4 | 7 |
|  | 3 | 11 | 19 |
|  | 4 | 21 | 36 |
|  | 5 (Completely agree) | 22 | 38 |
| 1. You were able to enter your drink intake correctly into the smartwatch | 1 (Completely disagree)  2 | 4 | 7 |
|  | 3 | 13 | 22 |
|  | 4 | 17 | 29 |
|  | 5 (Completely agree) | 24 | 41 |

#### How did you feel about having to charge the smartwatch every night?

This was a free-text response, the full responses can be seen in Table 7.

*Table S7* - participants' attitudes to charging the smartwatches (N=82).

| Malay | English | Count |
| --- | --- | --- |
| -99 | Refused to answer | 14 |
| Sangat selesa | very comfortable | 1 |
| Sangat baik | very good | 1 |
| Sangat mudah | very easy | 1 |
| best | best | 1 |
| Baik | Good | 3 |
| Mudah | easy | 2 |
| Mudah dicas | easy to charge | 1 |
| Seronok | Fun | 3 |
| Senang | Happy | 3 |
| senang | happy | 2 |
| Gembira, sebab boleh menggunakannya esok | happy because i can use it tomorrow | 1 |
| mudah | easy | 2 |
| SELESA | comfortable | 1 |
| Selesa | comfortable | 1 |
| baik | good | 1 |
| Baik dan mudah | good and easy | 1 |
| Agak mudah | quite easy | 1 |
| Ok | ok | 5 |
| Okey | ok | 1 |
| Okey je | it's ok | 1 |
| OK | ok | 1 |
| Okay je | it's ok | 1 |
| okay | ok | 1 |
| Biasa | normal | 1 |
| Biasa saja | it's normal | 1 |
| tiada perasaan | no feelings | 1 |
| Tiada | none | 1 |
| Tak selesa | uncomfortable | 1 |
| Susah sikit | a little difficult | 1 |
| Gembira | Easy | 1 |

#### Any other comments?

Additional comments and their translations can be seen in Table 8. Most participants left no comment.

*Table S8 - additional comments by the participants.*

| Malay | English | Count |
| --- | --- | --- |
| 5 | 5 | 1 |
| 6 | 6 | 1 |
| Selesa | Comfortable | 1 |
| Seronok | It's fun | 1 |
| tiada | no | 1 |
| tidak selesa dipakai kerana memakainya dalam jangka masa yang lama | uncomfortable to wear because of wearing it for a long time | 1 |
| Saya rasa sungguh risau apabila memakai jam aktiviti monitor ketika tidur kerana takut akan merosakkan jam tersebut akibat terketuk pada tiang katil. | I feel really worried when wearing the monitor activity clock while sleeping for fear of damaging the clock due to knocking on the bedpost | 1 |

### Smartwatch

Statistics on the median and IQR number of responses per day for all participants, including those who took part during Ramadan can be seen in Table 9. Statistics for all participants including catch-up entries can be seen in Table 10.

*Table S9* - a summary of smartwatch responses by day, for all participants including those who took part during Ramadan.

| Median (IQR) | Meal | Drink | Snack |
| --- | --- | --- | --- |
| Day 1 | 2.0 (3.0) | 2.0 (4.0) | 1.0 (2.0) |
| 2 | 2.0 (2.0) | 2.0 (4.0) | 1.0 (2.0) |
| 3 | 2.0 (2.0) | 2.0 (3.0) | 0.0 (1.0) |
| 4 | 2.0 (2.0) | 1.0 (3.0) | 0.0 (1.0) |
| 5 | 1.0 (2.0) | 1.0 (2.0) | 0.0 (1.0) |
| 6 | 1.0 (2.0) | 1.0 (3.0) | 0.0 (1.0) |
| 7 | 0.5 (2.0) | 0.0 (2.0) | 0.0 (1.0) |

*Table S10* - a summary of smartwatch responses by day for all participants, including catch-up events.

| Median (IQR) | Meal | Drink | Snack |
| --- | --- | --- | --- |
| Day 1 | 2.0 (3.0) | 2.0 (4.0) | 1.0 (2.0) |
| 2 | 2.0 (2.0) | 2.0 (4.0) | 1.0 (2.0) |
| 3 | 2.0 (2.0) | 2.0 (3.0) | 0.0 (1.0) |
| 4 | 2.0 (2.0) | 1.0 (3.0) | 0.0 (1.0) |
| 5 | 1.0 (2.0) | 1.0 (3.0) | 0.0 (1.0) |
| 6 | 1.0 (2.0) | 1.0 (3.0) | 0.0 (1.0) |
| 7 | 0.5 (2.0) | 0.0 (2.0) | 0.0 (1.0) |

The median of the mean response rate per day is shown in Table S611.

Table S61 - the median and interquartile range of response rates per day.

| Day | Median /% | IQR |
| --- | --- | --- |
| 1 | 83 | 67, 92 |
| 2 | 75 | 58, 92 |
| 3 | 75 | 50, 83 |
| 4 | 67 | 48, 83 |
| 5 | 75 | 42, 84 |
| 6 | 67 | 42, 83 |
| 7 | 58 | 33, 75 |
