# Supplementary material for: The Feasibility of Smartwatch Micro–Ecological Momentary Assessment for Tracking Eating Patterns of Malaysian Children and Adolescents in the South-East Asian Community Observatory Child Health Update 2020: Cross-Sectional Study
Source: J Med Internet Res. 2026 Feb 6;28:e73435. doi: 10.2196/73435 (PMC12924041; doi:10.2196/73435)
Supplement: Multimedia Appendix 2 [file jmir_v28i1e73435_app2.pdf]

CREMAS Checklist

Topic	Item	Section Reported	Notes
1. Title	Include ecological momentary assessment in title and key words	Title	
Introduction			
2. Rationale	Briefly introduce the concept of EMA and provide reasons for utilizing EMA for this study or topic of interests	Introduction	"Ecological Momentary Assessment (EMA) is the repeated sampling of current behaviours in real-time in a natural environment"
Methods			
3. Training	Indicate if, and by what methods, training of participants for EMA protocol was used	Methods	"Participants were briefed on the use of these devices by the data collectors including how to charge them and how to replace them after charging"
4. Technology	Describe what technology, if any, was used. Include the following information: device (eg, mobile phone, portable computer), model (eg, Nexus 4, iPod), operating system (eg, Android, Windows), and EMA program name	Methods	"participants were also given TicWatch C2 Android smartwatches".
5. Wave Duration	State the number of waves for the study (eg, 2 monitoring periods over the course of 1 year)	Methods	Single-wave study.
6. Monitoring period	State the number of days each wave of the study lasted, and how many weekdays versus weekend days	Methods	"worn 24 hours/day over 7 days". "Participants in this study began wearing the devices on different days of the week" mentioned in Discussion.
7. Prompting design	Indicate the prompting strategy used for the study (eg, event-based, interval-based, or a combination of the two). If using interval-based strategy, indicate what type of schedule is used (eg, fixed, random, or hybrid interval)	Methods	"prompts were scheduled to appear once every hour from 9am to 8pm"

8. Prompt Frequency	Intended frequency of prompts per day. Break down by weekdays and weekend days if applicable	Methods	"prompts were scheduled to appear once every hour from 9am to 8pm"
9. Design features	Describe any design feature to address potential sources of bias (eg, reactivity) or participant burden (eg, EMA questions appearing in different orders)	Methods	EMA protocol is described in the Methods, including the prompts, possible responses and functionality to go back to edit previous responses.
Results			
10. Attrition	Indicate participant attrition throughout the study; report attrition rates both by monitoring days and waves, if applicable	Results	Estimated in Figure 2.
11. Prompt delivery	Report number of EMA prompts that were planned to be delivered. If possible, also report the number of EMA prompts that were actually received by participants and indicate reasons for why prompts were not sent out (eg, technical issues or participant noncompliance reason such as phone was powered off)	Results	Planned number of prompts reported in Methods. Issues with receiving the prompts (e.g. battery issues) discussed in Discussion.
12. Latency	Report the amount of time from prompt signal to answering of prompt		Not recorded, but maximum latency (three minutes, after two reminder prompts) described in Methods.
13. Compliance rate	Report total answered EMA prompts across all subjects and the average number of EMA prompts answered per person. Report compliance rate both by monitoring days and waves, if applicable. Indicate reasons for noncompliance, if known	Results	Reported in Results; e.g. Table 2, Figure 3.
14. Missing data	Report whether EMA compliance is related to demographic or time-varying variables	Results	Time- and demographic-related trends reported in Results.
Discussion			
15. Limitations	Discuss limitations of the study, taking into account sources of potential bias when using EMA	Discussion	Discussed in "Strengths and Limitations" subsection.

	methods (eg, reactivity, use of technology)		
16. Conclusions	Provide a general interpretation of results and discuss the benefits of using EMA	Discussion, Conclusion	General interpretation given in Conclusions; EMA discussed in Discussion.
